# No observation of DIANA signals in rats at 7.0 and 17.2 Tesla

**DOI:** 10.1162/imag_a_00136

**Published:** 2024-04-18

**Authors:** Martijn A. Cloos, Erwan Selingue, Shota Hodono, Romain Gaudin, Luisa Ciobanu

**Affiliations:** Australian Institute for Bioengineering and Nanotechnology, University of Queensland, St. Lucia, Australia; NeuroSpin/CEA, University Paris-Saclay, Gif-sur-Yvette, France

**Keywords:** functional MRI, neuronal activity, DIANA, physiological noise, preclinical MRI

## Abstract

Recently, a new method was introduced to detect neuronal activity using Magnetic Resonance Imaging (MRI). The method, referred to as DIANA, showed MRI signals with millisecond temporal resolution that correlated with local field potentials measured invasively in mice. Troublingly, attempts by other groups to detect the DIANA signals in humans at 7 Tesla and mice at 15.2 Tesla have failed. So far, attempts to reproduce DIANA in small rodents have focused on paradigms using whisker pad stimulation, which were expected to produce a 0.1–0.15% signal change. However, the Supplementary Material accompanying the original DIANA paper showed that visual stimulation produced a three times larger signal, which should be much easier to detect. Therefore, we attempted to find the DIANA signal in rats using a visual stimulation paradigm. Experiments were performed at 17.2 Tesla but also at 7.0 Tesla to see if the DIANA signal appears at a lower field strength where T2 is longer and BOLD contributions are reduced. In addition, simulations were performed to investigate the theoretical detectability of synthetic DIANA signals in noisy data. Although our data indicated that a 0.1% signal change would have been detectable, we did not observe a DIANA signal. We did observe neuronally driven hemodynamic signal variations that were much larger than the anticipated DIANA signal. The amplitude of these signal changes was relatively similar at 7.0 and 17.2 Tesla (0.7% vs 1.1%). Numerical simulations indicated, however, that the measured hemodynamic signal changes would not interfere with the detection of DIANA signals. Therefore, it is reasonable to expect that measurements at higher field strength with improved SNR would have a better chance to detect the DIANA signal. Yet, we, among others, were unable to find it.

## 1 Introduction

Functional Magnetic Resonance Imaging (fMRI) has become a cornerstone of modern-day neuroscience. Conventional fMRI techniques infer neuronal activation from associated hemodynamic changes (Huber et al., 2017;Kim & Ogawa, 2012;Logothetis, 2008;Logothetis et al., 2001;Ogawa et al., 1990). As such, these fMRI methods are biased by the architecture of the vascular network (Chaimow et al., 2018;Menon, 2012;Polimeni et al., 2010;Turner, 2002) and the complex mechanisms associated with neurovascular coupling (Buxton et al., 2004;Havlicek & Uludağ, 2020;Hillman, 2014). Although surprisingly rich in information, these hemodynamic signals thus paint a distorted picture of the underlying neuronal activity.

Toi et al. (2022)published a method that aims to directly image neuronal activity using MRI. Their method, called DIANA, showed MRI signals with millisecond temporal resolution that correlated with local field potentials measured invasively in mice. Arguably, such a method would be the most impactful when applied to human imaging where electrophysiological measurements are normally impossible. However, initial attempts to translate DIANA from mice to humans have failed (Hodono et al., 2023).

TroublingChoi et al. (2023)were unable to reproduce the DIANA signal in mice. Their experiments closely followed the methods proposed byToi et al. (2022)but deviated on two points. First, Choi et al. used a stronger magnet, 15.2 Tesla instead of 9.4 Tesla. Stronger magnets produce a larger MRI signal, increasing the Signal to Noise Ratio (SNR). However, stronger magnetic fields also enhance Blood Oxygenation Level Dependent (BOLD) signal (Ogawa et al., 1990;Uludağ et al., 2009) and certain forms of physiological noise such as breathing-related magnetic field variations (Moortele et al., 2002). Both of these effects could obscure small non-hemodynamic neuronal signals, if they exist. Second,Choi et al. (2023)used continuously infused sedation, instead of intermittent injections, providing more stable anesthesia and allowing long, continuous, measurements. Although this is more efficient, the authors of the original DIANA paper suggested it might be better to conduct many short measurements (Park, 2023), giving the animal a break in between measurements, similar to the work byToi et al. (2022).

In this work, we attempted to find the DIANA signal in rats. The manuscripts by Toi et al. and Choi et al. focus on results obtained using electrical stimulation of the whisker pad. However, supplementary material accompanyingToi et al. (2022)showed that visual stimulation produced a much larger DIANA signal (Figure S26inToi et al. (2022)), 0.5% versus 0.15%. Therefore, we opted for visual stimulation. Experiments were performed at 17.2 Tesla but also at 7 Tesla to see if the DIANA signal appears at a lower field strength, where T2 is longer and BOLD contributions are reduced. Moreover, we collected data under two different anesthetic regimes: medetomidine, a popular choice for fMRI studies in rats (Weber et al., 2005), and ketamine/xylazine, the anesthetic used by bothToi et al. (2022)andChoi et al. (2023). After characterizing the temporal signal to noise ratio (tSNR) and hemodynamically driven background signal variation, simulations were performed to investigate the theoretical detectability of a DIANA response.

## 2 Methods

### 2.1 Animal preparation

Eleven female Sprague Dawley rats were used in this study. Among these, seven animals were anesthetized with medetomidine (0.05 mg/kg subcutaneous bolus followed by 0.1 mg/kg/h continuous subcutaneous infusion) and were scanned at 7 Tesla and/or at 17.2 Tesla: one animal was scanned at both 7 and 17.2 Tesla, three only at 17.2 Tesla, and three only at 7.0 Tesla (seeTable S1for details). Four animals were anesthetized with ketamine/xylazine following the protocol in (Hutchison et al., 2010), that is, 80/10 mg/kg bolus followed by 50/6 mg/kg continuous intraperitoneal infusion and were scanned only at 17.2 Tesla. In both anesthesia protocols, the animals were spontaneously breathing oxygen-enriched medical air. For all animals, the experimental time was ~2 hours. The DIANA acquisitions (see below) started 30 to 45 min after the onset of anesthesia infusion and lasted 51 to 56 min, depending on the number of repetitions. The body temperature of the animals was maintained in the range of 36–37.5°C using a circulating hot water blanket. An optical fiber was placed in front of the left eye. The fiber was connected to a blue LED controlled by an Arduino (ArduinoCham, Switzerland). The internal clock of the Arduino was referenced to determine the stimulus duration and timing within the trial. The stimuli were synchronized with the scanner using Transistor-Transistor-Logic (TTL) signals. The same stimulation setup was used in both MRI systems. All animal procedures were approved by the Comité d’Ethique en Expérimentation Animale, Commissariat à l’Energie Atomique et aux Énergies Alternatives, and by the Ministère de l’Education Nationale, de l’Enseignement Supérieur et de la Recherche (France) under reference #33846-2021110918272220 and were conducted in strict accordance with the recommendations and guidelines of the European Union (Directive 2010/63/EU) and the French National Committee (Décret 2013–118).

### 2.2 Experimental setup and acquisition parameters

Experiments were performed using 17.2 (housed in a shielded room) and 7.0 Tesla (housed in an unshielded room) MRI systems (Bruker BioSpin, Erlangen, Germany). At 17.2 Tesla, a 2 cm TR/RX surface coil (RAPID Biomedical GmbH, Rimpar, Germany) was used. At 7 Tesla, a receive-only 2 × 2 array coil was used in combination with a 7.2 cm ID transmit volume coil (Bruker BioSpin, Erlangen, Germany).

#### 2.2.1 Slice placement & ROI preparation

At the start of each session, multi-slice gradient echo echo-planar imaging (GRE-EPI) functional BOLD acquisitions were performed to identify the best slice for single-slice acquisitions (TR = 1000 ms, TE@17.2 Tesla = 10 ms, TE@7 Tesla = 16 ms, spatial resolution = 200 × 200 × 800 μm^3^, slices = 5, FA = 50°, volumes = 372). Five coronal slices were positioned 7.2 to 3.2 mm posterior to bregma, covering the visual cortices and the superior colliculus (SC). The visual paradigm consisted of 6 s LED-on (flashing at 2 Hz) and 12 s LED-off. The data were analyzed online, using the image processing tools included with the MRI system (Paravision 6, Bruker BioSpin, Erlangen, Germany). The slice with the most activation was used for all subsequent measurements.

BOLD weighted single-slice Spoiled Gradient Recalled Echo (SPGRE) experiments were performed (TR = 25 ms, TE@17.2 Tesla = 10 ms, TE@7 Tesla = 16 ms, 80 x 80 matrix, 250 × 250 × 1500 μm^3^, FA = 8°@17.2 Tesla, 9°@7 Tesla, 180 volumes) with normal k-space ordering to obtain BOLD-based activation maps without EPI distortion. The stimulation paradigm consisted of 4 s LED-on (flashing at 2 Hz) and 10 s LED-off.

The data were reconstructed and analyzed in Matlab (MathWorks, Natick, MA, USA) using SPM12 (https://www.fil.ion.ucl.ac.uk/spm/). Functional Regions of Interest (ROI) were drawn using ITK-SNAP (Yushkevich et al., 2006), selecting only voxels with a t-score of 2.5 or higher. Control ROIs were drawn in the midbrain reticular nucleus. In each rat, the functional and control ROIs had a matching number of voxels to ensure similar statistics.

#### 2.2.2 DIANA measurements

**Fig. 1.**
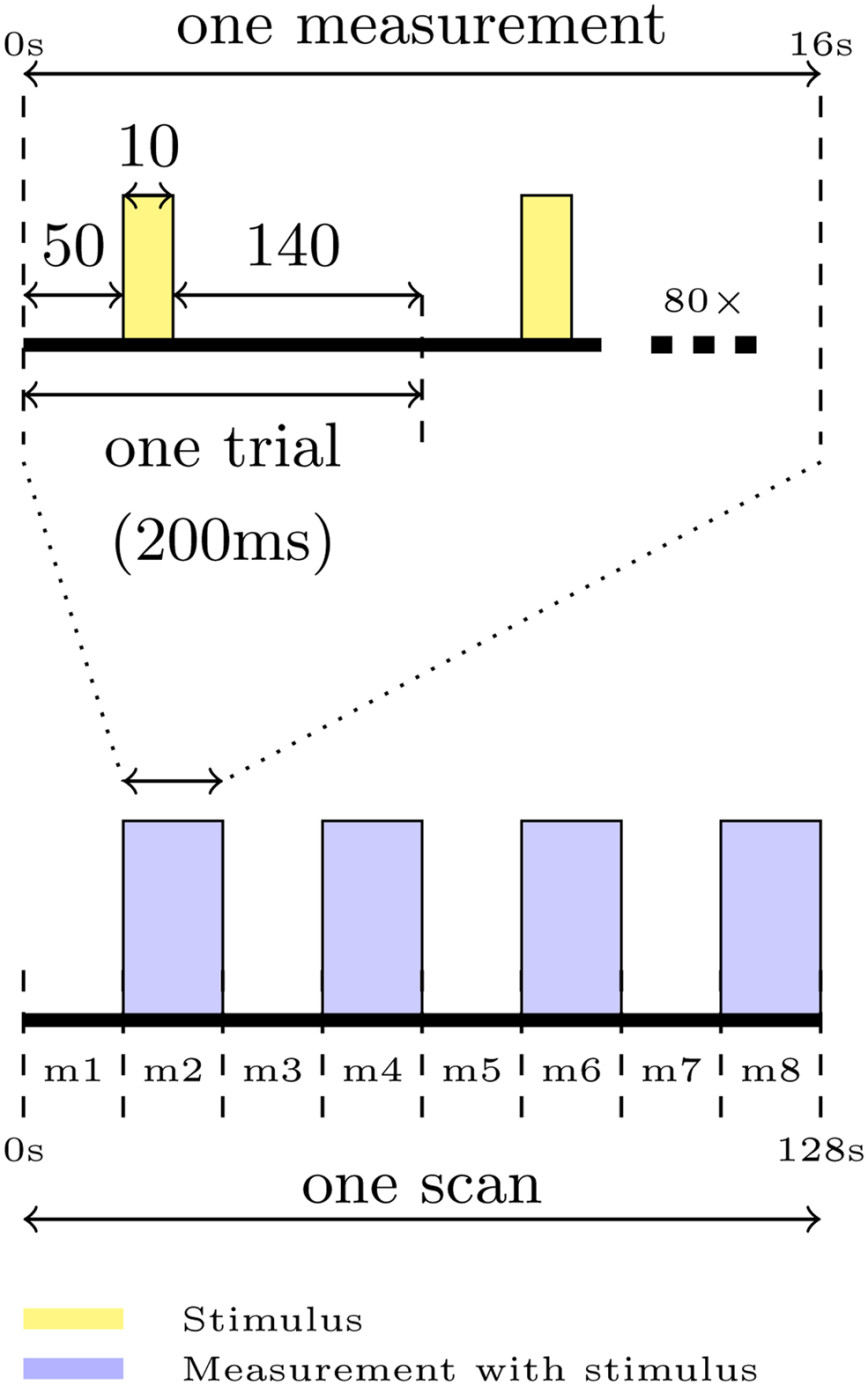
Overview of the DIANA paradigm used in this study. All DIANA experiments used 200 ms long trials. A 10 ms visual stimulus was presented after 50 ms, followed by a 140 ms delay until the end of the trial. The trial was repeated 80 times to collect all phase encoding lines needed to construct a complete set of 40 fully sampled images (5 ms effective temporal resolution). The process of collecting 40 fully sampled time points constitutes one DIANA measurement. Eight DIANA measurements were collected in each scan. The stimulus was on during even numbered measurements only.

All DIANA experiments used a modified version of the product SPGRE sequence in which the phase and measurement loops were swapped (TR/TE = 5/1.8 ms, 80 x 80 matrix, 250 × 250 × 1500 μm^3^, FA = 6°) (Silva & Koretsky, 2002). To stabilize the magnetization, 3200 dummy TRs were played at the start of each scan (Brown et al., 2014;Hodono et al., 2023). Forty DIANA time points were collected per phase encoding step and repeated 80 times to fill all of k-space (16 s per measurement). Eight measurements were collected per scan (2 min 24 s). For each scan, the stimulus was kept off during odd numbered measurements (Fig. 1). Breaks of ten seconds were allowed between two consecutive DIANA measurements.

To synchronize the stimulus, the sequence was programmed to send a TTL signal every TR. The stimulation consisted of 10 ms LED-on and 190 ms LED-off (Fig. 1). Phantom experiments were performed to validate the stability of the signal (50 ml falcon tube filled with 2% agarose doped with CuSO_4_and containing three small LEGO bricks, T2 ~ 60 ms at both field strengths). In addition, the gradient waveforms produced by the scanner were recorded using an oscilloscope (LeCroy WavePR0 7100A, Teledyne, Heidelberg, Germany) to validate the timing of sequence. These measurements revealed that the effective TR was ~5.15 ms, therefore longer than the nominal TR of 5 ms. However, we did not see a delay accumulation during our 2 min 24 s experiment. From the stimulus point of view, the fact that the TR is effectively longer than 5 ms does not pose a synchronization problem as we trigger every TR. From a spin-dynamics point of view, the TR was constant, which means the effectiveness of gradient and RF spoiling was not compromised. For simplicity, the results in the manuscript (including figures) are presented as if the TR was exactly 5 ms.

In the medetomidine anesthesia group, 92 scans were collected across four rats, for a total of 372 and 368 pairs of stimulus on stimulus off measurements at 7.0 and 17.2 Tesla, respectively. In the ketamine/xylazine group, 88 scans (352 pairs of stimulus on stimulus off measurements) were acquired at 17.2 Tesla across four rats. An overview can be found inTable S1.

### 2.3 Data analysis

#### 2.3.1 DIANA data analysis

All DIANA data were reconstructed and analyzed in Matlab (MathWorks, Natick, MA, USA). The DIANA timeseries was processed in three ways.

All DIANA data were visually screened for possible outside RF interference in the data. Eight measurements collected at 7.0 Tesla were removed, four with and four without stimulus.

To investigate if a DIANA signal was present, each scan was cut into individual measurements. Measurements were sorted into two batches, with and without stimulus. Each batch was processed the same way. The signal was converted to percent signal change, detrended (linear, using Matlab’s build in function: ‘detrend’). Smoothing was applied along the time domain using a gaussian filter (width = 3 and sigma = 1), assuming cyclic boundary conditions. The data were then averaged across voxels in the ROI, measurements, and rats.

To study signal changes over long timescales, the signal collected in each scan was converted to a percent signal change (voxel by voxel) and averaged across voxels in the ROI. At 7 Tesla, the shim coils were heated up during the scan leading to frequency drifts. Although these effects were approximately linear across the time span of an individual measurement (16 s), non-linear B0 changes were observed over the duration of a complete scan. Therefore, the signal was detrended using a 6^th^order polynomial, fitted to the control ROI data. This fit was then used to detrend both the functional and control ROI data. The detrended signals were averaged across scans and plotted as a function of time. At 17.2 Tesla, the shim coils were temperature controlled, resulting in minimal B0 drift. For consistency, all data were processed the same way.

#### 2.3.2 tSNR assessment

To estimate the sensitivity of the measurements, the tSNR in each ROI was calculated for each measurement. First, the signal was averaged across voxels in the ROI. The tSNR was then calculated as the mean signal over the standard deviation of the signal across all 40 time points in each measurement. The average tSNR across all odd (stimulus off) and all even (stimulus on) measurements was recorded separately. In addition, for each rat, the tSNR was plotted as a function of the number of measurements (N). In these plots, each data point represents the average tSNR found by randomly picking 80 combinations of N measurements out of the pool of all measurements obtained during that scan session.

#### 2.3.3 Simultaneous DIANA and BOLD fMRI

When using short ISI, the BOLD signal eventually settles into a steady-state plateau with small signal variations on top (Lewis et al., 2016). Although DIANA measurements use a small TE, some residual BOLD, Cerebral Blood Volume (CBV), and Cerebral Blood Flow (CBF) changes are likely to shine through. Therefore, we expected to see a difference in signal amplitude between even (stimulus on) and odd (stimulus off) DIANA measurements. To validate this hypothesis, we averaged all scans (each containing eight measurements) and plotted the percentage signal change over time in each ROI. BOLD fMRI maps were constructed using SPM12 (https://www.fil.ion.ucl.ac.uk/spm/). In these analyses, the functional signal was modeled as a boxcar (0 during odd measurements, 1 during even measurements). Groups of three consecutive sets (~6 min of data) were averaged to improve the statistics. No smoothing or clustering was applied.

#### 2.3.4 Hemodynamic background assessment

To investigate the impact of hemodynamic signal variations on DIANA experiments, we also obtained data using single-slice BOLD weighted gradient echo EPI (TR = 50 ms, TE = 10 ms, 80 × 80 matrix, 250 × 250 × 1500 μm^3^, FA = 8°, 2560 volumes). DIANA experiments used a short TE, which could be weighted more towards CBV and CBF effects (Kim & Ogawa, 2012;Lu et al., 2005;Roy & Sherrington, 1890). Therefore, we also collected SPGRE images with traditional phase encoding (TR/TE = 5/1.8 ms, 80 x 80 matrix, 250 × 250 × 1500 (μm)^3^, FA = 6°. 0.4 s per volume). These measurements are listed inTable S1as “SPGRE*” and “EPI*”, not to be confused with the multi-slice EPI measurements and the BOLD weighted SPGRE used during slice placement and ROI delineation. Data from these measurements were analyzed the same way as the DIANA data.

### 2.4 Numerical simulations

#### 2.4.1 Simulation model

Simulations were performed to assess the detectability of synthetic DIANA signals. The baseline signal was constructed by averaging DIANA measurements from one of the odd numbered experiments (stimulus off). The BOLD SPGRE ROI, used to evaluate the experimental DIANA data, was used to create two images: One holding the signal in the ROI only ($S_{1}$) and one with zeros inside the ROI ($S_{2}$). These images were Fourier transformed to obtain their k-space contributions ($K_{1}$and$K_{2}$). Synthetic DIANA data were created by selecting lines in k-space according to the sampling patterns specified in the sequence. Eachnth synthetic readout ($K_{s,n}$) summed signal contributions according to:

$$K_{s,n}\left(t\right)=K_{2,n}+\beta \left(t\right)\Gamma \left(t\right)K_{1,n}+\sigma ,$$

whereβ(t)describes hemodynamic signal changes,Γ(t)describes the DIANA signal, andσdenotes additive complex gaussian noise. In our simulations,Γ(t)followed the timing shown in (Fig. 1), producing a 10 ms long 0.5% signal increase starting 50 ms after the stimulus ends.

#### 2.4.2 Simulation: DIANA with noise

Synthetic DIANA data sets were created based on the tSNR values estimated at 17.2 Tesla. In total, 360 measurements were created with additive complex noise. In these stimulations, hemodynamic signal variations were excluded (β(t)=1). For reference, one data set without noise was also created. The synthetic data were processed identically to the in-vivo data. Plots were created averaging 1, 30, 180, and 360 measurements.

#### 2.4.3 Simulation: DIANA with hemodynamics

Synthetic DIANA data sets were created with hemodynamic signal variations (β(t)). These variations were derived from the signal evolutions measured in-vivo using the SPGRE* sequence without DIANA ordering (TR/TE matching the DIANA experiment, 0.4 s temporal resolution). To exclude noise from these estimates, the measuredβ(t)were filtered using a gaussian filter of width 5. The estimates forβ(t)were interpolated (interp1 in Matlab using the ‘spline’ option) to a 5 ms temporal resolution. No gaussian noise was added to any of these simulations (σ=0). In addition, simulations were performed using sinusoidal background signals with 0.5% peak-to-peak amplitude. Frequencies of 0.5, 1, and 5 Hz were tested. A random phase shift was added to the start of each synthetic scan. To visualize the effect in image space, simulations were also performed with K_2_set to zero. The synthetic data were processed using the same tools used to process the in-vivo data.

## 3 Results

### 3.1 Experiments

The results presented in this section are obtained under the medetomidine anesthesia protocol. The results obtained under ketamine/xylazine are reported in theSupplementary Information.

**Fig. 2.**
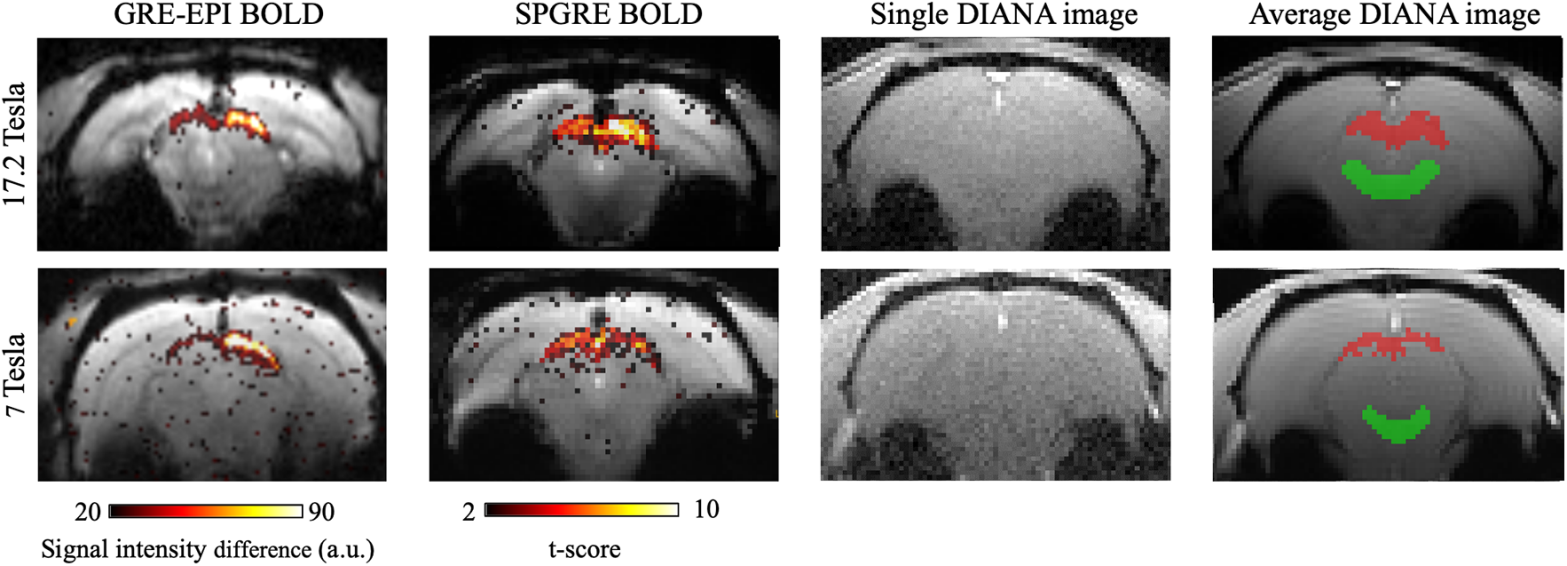
Functional localization and ROIs. The leftmost column shows the functional maps (signal intensity difference) created at the scanner using GRE-EPI BOLD. The second column from the left shows the distortion marched t-score maps obtained using the SPGRE BOLD protocol. The third column from the left shows single DIANA images. The rightmost column shows exemplary ROIs drawn on average (n = 20 images) DIANA images. Examples taken from rat 4 at 17.2 Tesla, top row and 7 Tesla, bottom row.

Figure 2shows exemplary t-score maps obtained using the GRE-EPI BOLD functional localizer (leftmost column), the SPGRE BOLD measurement in the target slice (second column from the left), and a representative DIANA image with the SPGRE-BOLD based ROI superimposed in red (rightmost column). The green ROIs inFigure 2were used as control areas for the analysis of signal variations across long time scales. Activated and control ROIs contained the same number of voxels.

**Fig. 3.**
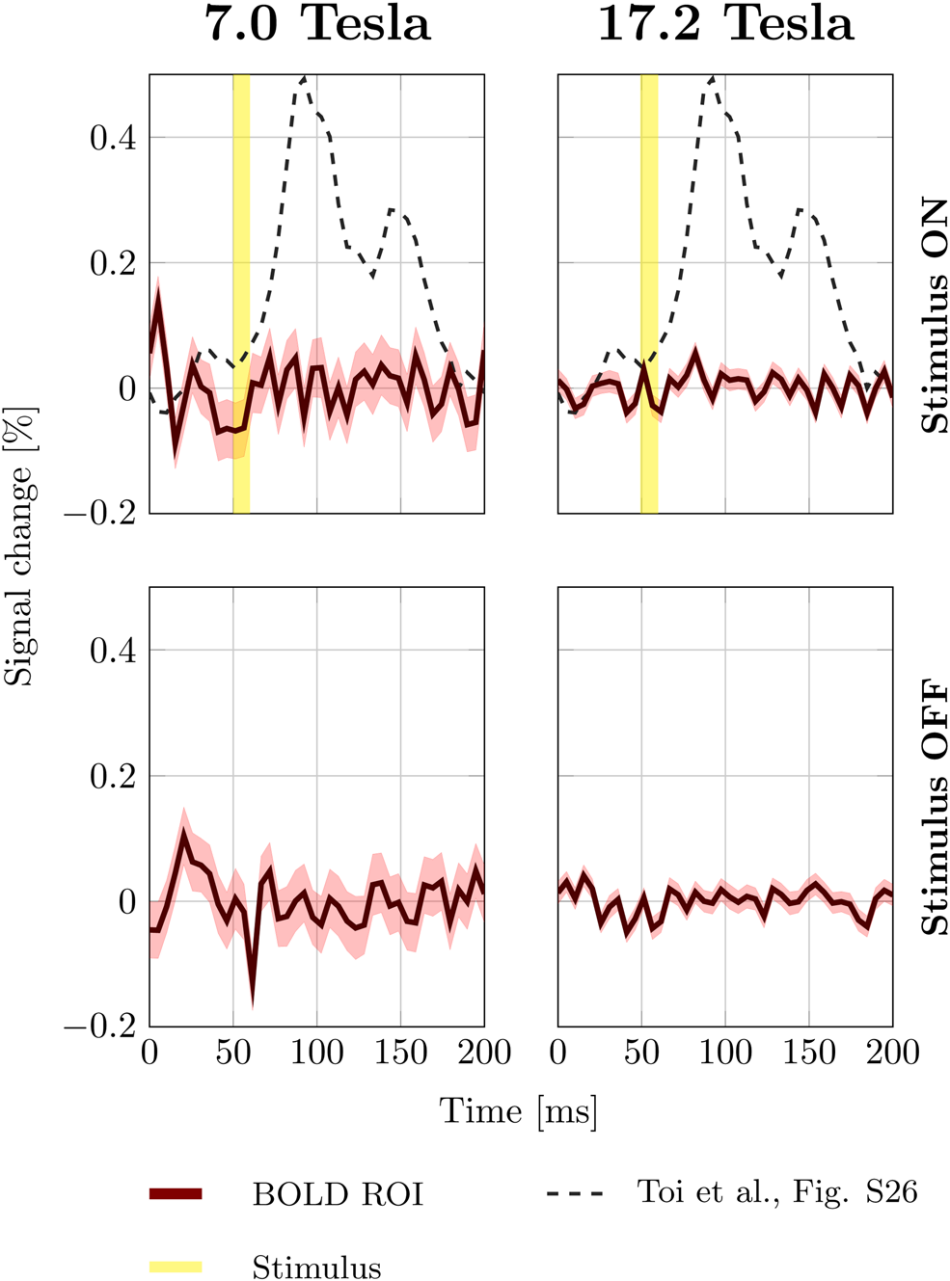
Trial averaged signal evolution observed in the BOLD ROI. Each plot contains data from four rats. In total, 368 measurements were averaged. Solid lines indicate the mean signal across measurements. Shaded areas indicate the 99% confidence interval. The dashed line shows the expected DIANA signal based onFig. S26in Toi et al. The top row shows the signal measured with the stimulus on. The yellow vertical line represents the stimulus. The bottom row shows the signal measured without stimulus.

Figure 3shows the trial averaged DIANA signals measured at 7.0 and 17.2 Tesla (four rats per magnet, 368 measurements in total). The top row shows the trial averaged signal in the BOLD ROI with the stimulus on. No distinct signal peak was observed at either field strength. For comparison, the expected DIANA signal, based onFigure S26in Toi et al. (Toi et al., 2022), is shown in the background (dashed). The bottom row shows the trial averaged signal measured combining 368 measurements without stimulus. The signals collected with and without stimulus look very similar.

**Fig. 4.**
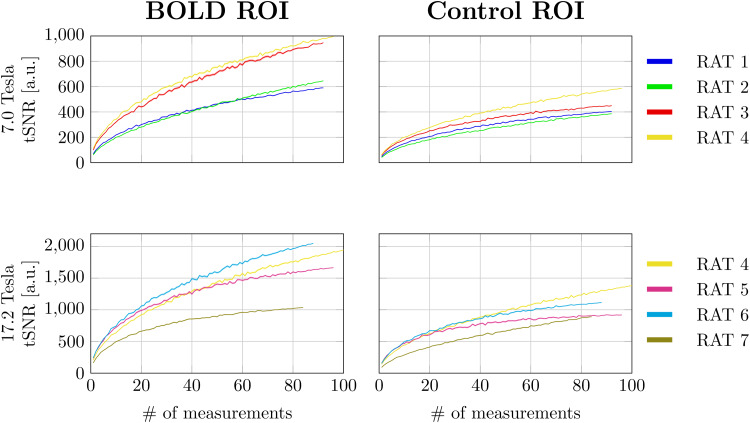
The tSNR as a function of the number of measurements. Solid lines indicate the mean signal across measurements. The shaded areas indicate the 99% confidence interval. Note that the confidence interval is very narrow and difficult to see.

The time normalized tSNR estimates are summarized inTable S2. The tSNR did not change significantly between measurements with and without stimuli, indicating that the signal variations detected with and without stimulus were nearly identical.Figure 4shows the tSNR as a function of the number of measurements. In all animals, the tSNR improved proportional to the square root of the number of measurements.

**Fig. 5.**
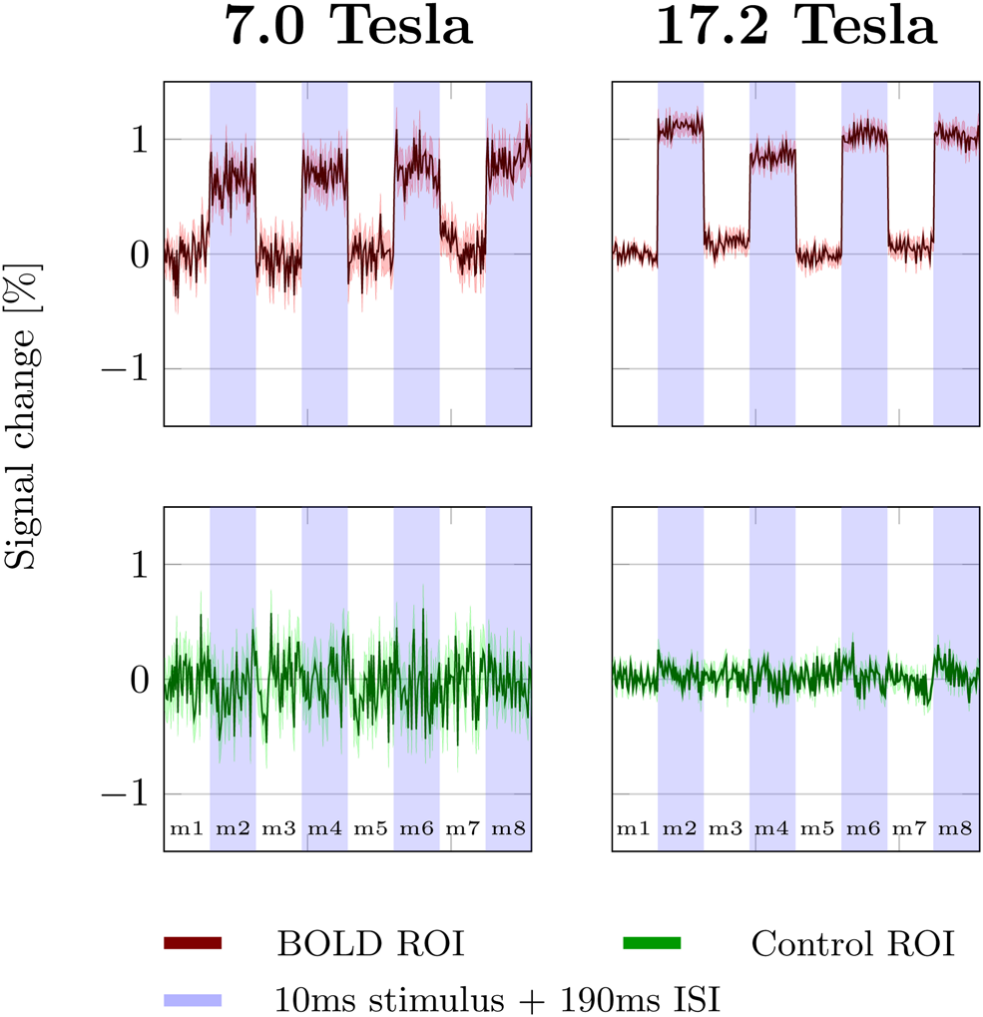
Average signal evolution observed throughout the scans. Each scan contained eight subsequent DIANA measurements (128 s of data). The stimulus was on during even numbered measurements only. Solid lines indicate the mean signal across measurements. Shaded areas indicate the 99% confidence interval. The control ROI was placed deeper in the brain with lower tSNR (Table S1).

Figure 5shows the average time course of the signal in the BOLD (red) and control (green) ROIs throughout the scan. When the stimulus was on, the signal increased by 0.7% at 7 Tesla and 1.1% at 17.2 Tesla.

**Fig. 6.**
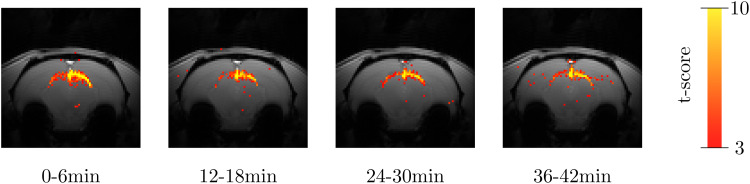
Functional activation maps derived from hemodynamic signals measured simultaneously during DIANA experiments. Activation was detected consistently in the superior colliculus throughout the scan session. Exemplary data from rat 4 at 17.2 Tesla.

The hemodynamic signal change observed between measurements with and without stimulus was used to construct functional maps (Fig. 6). Functional maps derived from hemodynamic functional signals measured simultaneously during the DIANA measurements showed that activation remained consistent throughout the DIANA scan sessions.

**Fig. 7.**
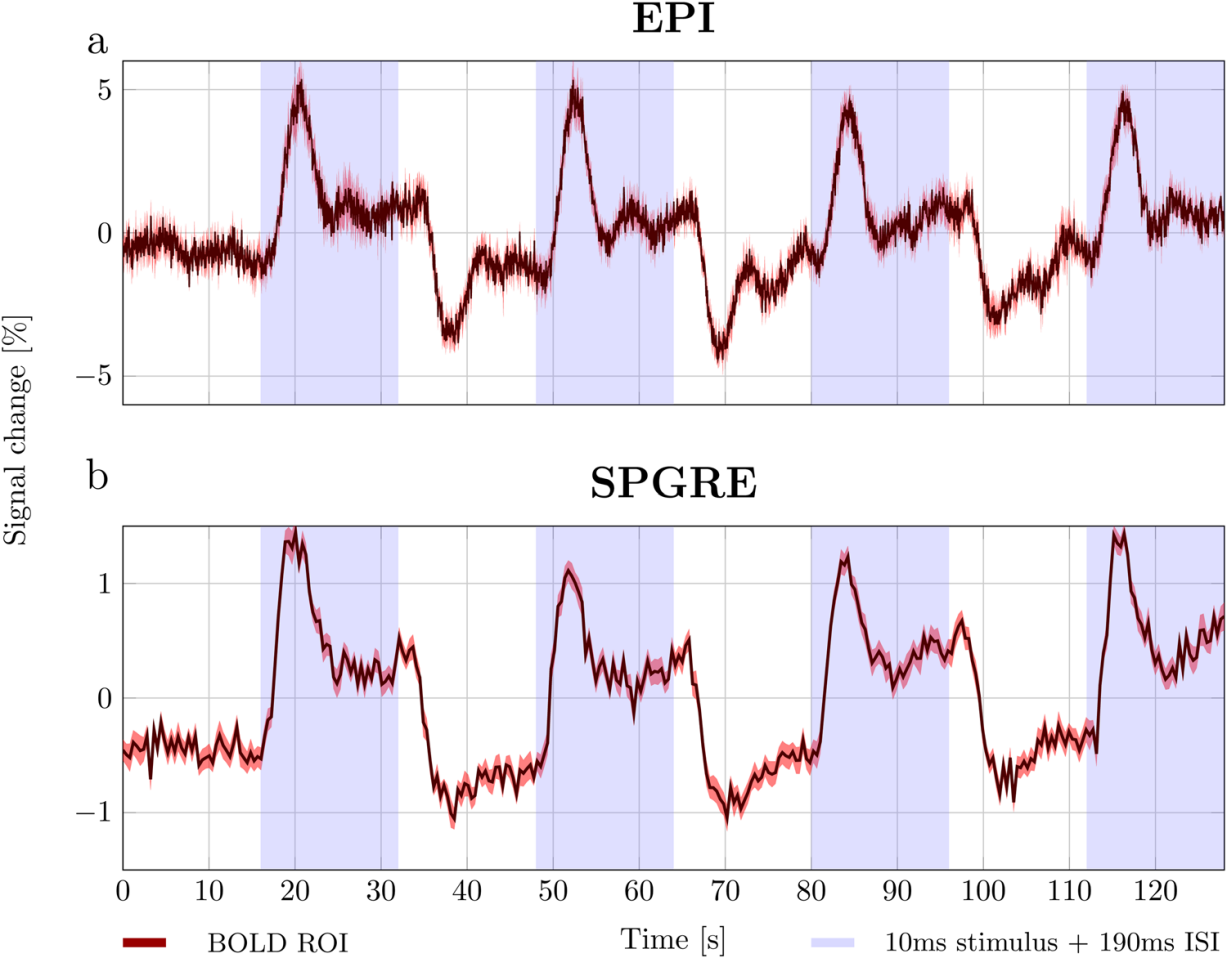
Signal changes observed using EPI and SPGRE. The stimulus (10 ms light flash 190 ms ISI) was only on during the shaded areas. (a) BOLD weighted GRE-EPI signal evolution (TR/TE = 50/10 ms). (b) SPGRE signal evolution. Solid lines indicate the mean signal across measurements (TR/TE = 5/1.8 ms, 0.4 s temporal resolution). Shaded areas indicate the 99% confidence interval.

Figure 7shows signal changes measured at 17.2 Tesla using single-slice GRE-BOLD EPI* (TR/TE = 50/10 ms, 50 ms temporal resolution) and SPGRE* measurements (TR/TE = 5/1.8 ms, 0.4 s temporal resolution), averaged across scans. GRE-BOLD EPI* measurements showed ~8% peak-to-peak signal variation. In general, the signal resembled BOLD signals commonly seen in block paradigms (Fig. 5). Following an initial peak, the BOLD signal settles in to a steady state. After the stimulus is turned off, the BOLD signal undershoots the baseline before it returns to equilibrium. However, the signal is never perfectly stable, showing signs of oscillations around 0.1–0.3 Hz. Despite much less T2* weighting, SPGRE* measurements (TE = 1.8 ms) still showed a clear hemodynamic signal (Fig. 7b), albeit with a reduced peak-to-peak amplitude (~1.5%).

ComparingFigures 5and7, we see that the DIANA measurement ‘washes away’ the dynamics of the hemodynamic signal. This is caused by the swapping of phase and repetition loops. In the DIANA measurement, the average signal is weighted towards the signal in the middle of the measurement where the center of k-space is sampled. As phase encoding lines are collected from k-max to k-min, the center of k-space is sampled at the 40^th^repetition of the trial, 8 s into the paradigm when the hemodynamic response has just passed its peak. Consequently, the dynamics of the hemodynamic signal are hidden, showing only discontinuous jumps in signal between DIANA measurements with and without stimulus.

### 3.2 Simulations

**Fig. 8.**
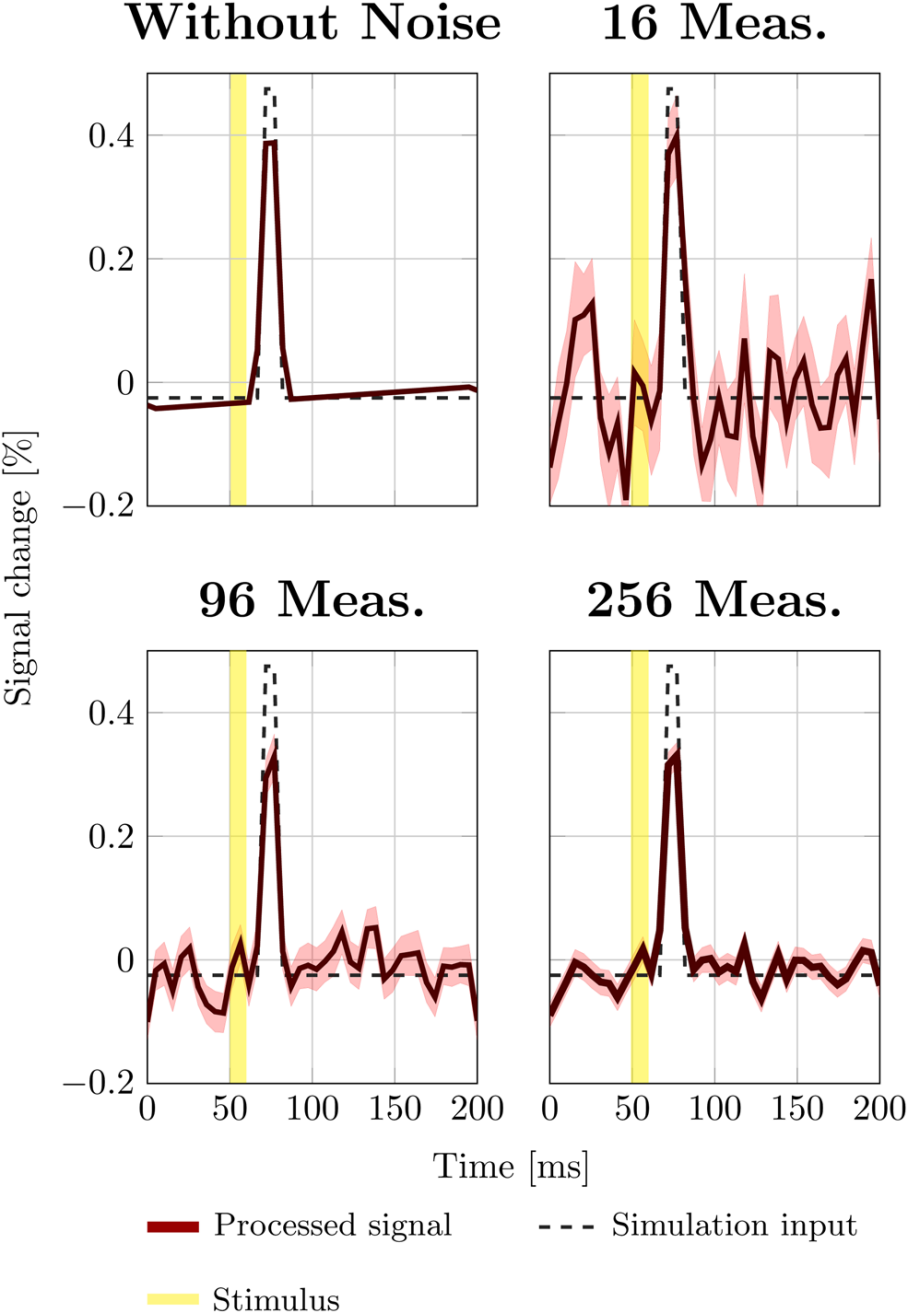
Simulated trial averaged signal evolutions. The noise level per measurement was matched to the tSNR observed in-vivo at 17.2 Tesla. Solid lines indicate the mean signal across simulated measurements. Shaded areas indicate the 99% confidence interval.

Simulations were performed using tSNR estimates derived from measurements performed at 17.2 Tesla (Table S1).Figure 8shows a simulated DIANA signal (0.5% signal change, 10 ms duration). Averaging as few as 16 measurements, a 0.5% DIANA signal can be seen above the noise. After averaging 96 measurements, even a 0.2% DIANA peak would have been visible. Averaging 256 measurements, comparable to cumulative data collected in four rats, the synthetic DIANA peak becomes clearly visible, with sufficient SNR to see DIANA signals as small as 0.1%.

The top row inFigure 9shows trial averaged synthetic DIANA experiments combining 23 scans containing eight DIANA measurements each. The stimulus was on only during even numbered measurements. The left plot shows the signal obtained using the hemodynamic background estimated from the average SPGRE* scans shown inFigure 6. Although the hemodynamic signal fluctuations were much larger than the synthetic DIANA signal (0.5%), the trial averaged response still showed a near-perfect clear DIANA peak. Simulations using a 0.5% (peak-to-peak) 1 Hz background signal indicated that these fluctuations were still too slow to contaminate the trial averaged synthetic DIANA signal in the ROI (middle column). The top right plot shows synthetic DIANA signals with a 0.5% (peak-to-peak) 5 Hz background signal. When using a 200 ms ISI, background fluctuations of 5 Hz and higher can produce oscillations in the trial averaged signal, modulating the confidence interval of the measurement.

**Fig. 9.**
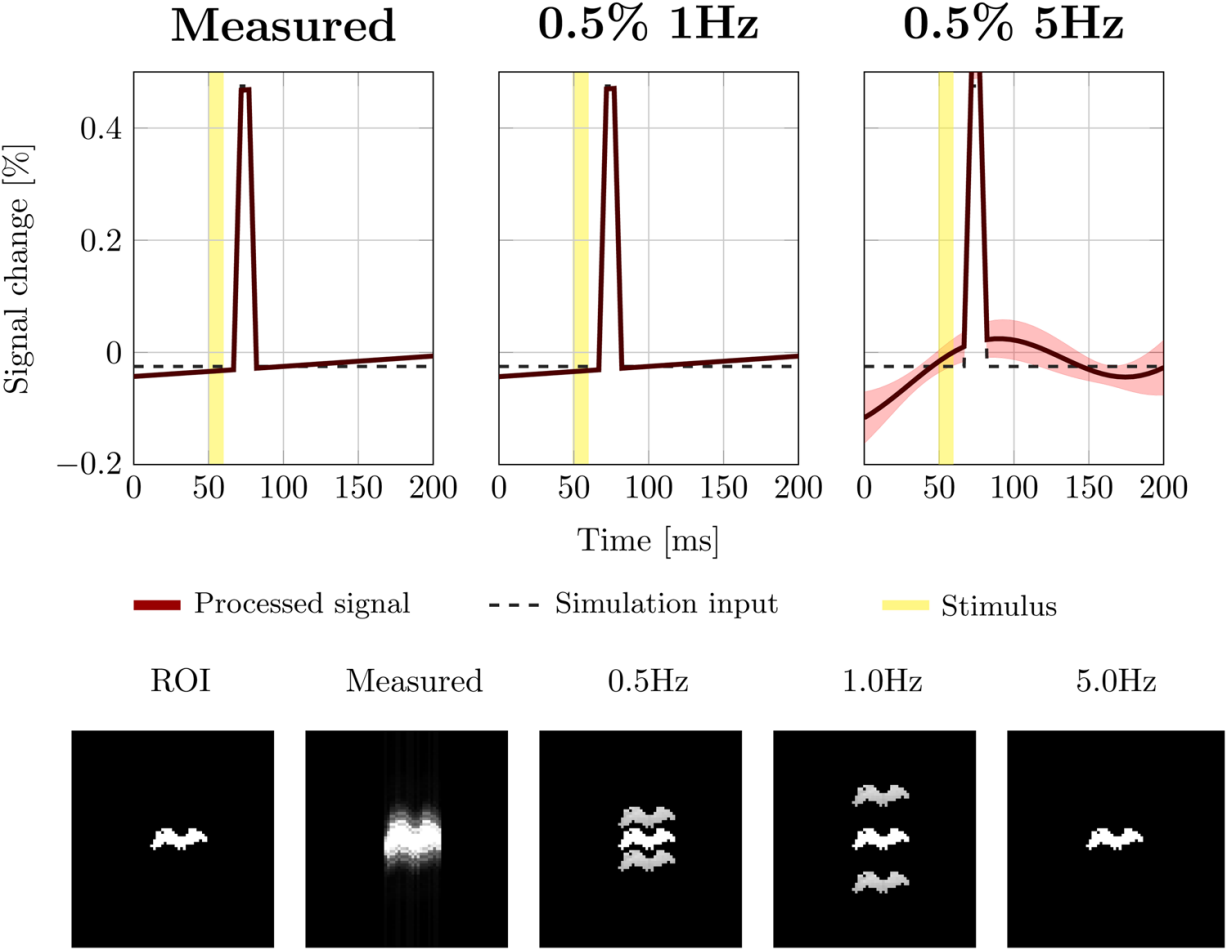
The top row shows simulated trial averaged signal evolutions with different hemodynamic background signals. From left to right, the panels show the synthetic signals obtained as follows: using hemodynamic signal variations measured with the SPGRE sequence (Fig. 5), using a 0.5% peak-to-peak 1 Hz background signal, and using a 0.5% peak-to-peak 5 Hz signal. Solid lines indicate the mean signal across simulated measurements. Shaded areas indicate the 99% confidence interval. The bottom row shows ghosts in image space produced by inconsistencies in k-space. The inverted phase and repetition loops can lead to an amplitude modulation along the phase encoding direction. From left to right: the ROI (ground truth), ghosts due to measured hemodynamic signal changes, and ghosts due to background signals at 0.5 and 1.0 Hz. Because the 5 Hz background signal synchronizes with the paradigm, the ‘ghosts’ land back on top of the ROI such that no image artifacts are produced.

Although slow signal modulations (<5 Hz) did not contaminate the trial averaged signal, they did lead to ghosts in image space (Fig. 9, bottom row). The distance between the mean image and ghosts depends on the frequency of the background dynamics relative to the ISI. The hemodynamic background variations estimated based on average SPGRE* scans contain a range of low frequencies that produce multiple ghosts with small shifts relative to the actual area of activation, thus blurring these signals in space. Using synthetic background fluctuations at isolated frequencies, these ghosts can be seen more clearly. Only when the frequency of the background fluctuation matches the stimulation frequency do the ghosts disappear. For example, a 5 Hz background fluctuation does not produce any blurring or artifacts. Because the 5 Hz background signal is synchronized with the paradigm (200 ms ISI), the ‘ghosts’ land back on top of the original signal location (bottom row, right most panel).

## 4 Discussion

We have attempted to detect DIANA signals in rats using 7.0 and 17.2 Tesla MRI using both medetomidine (shown in the main text) and ketamine/xylazine (Supplementary Figs. S1 and S2). Despite our best efforts, we were unable to identify neuronally driven signal contributions. However, we did observe neurovascular signal variations that were much larger than the anticipated DIANA signal (Fig. 4).

Whereas the manuscripts by Toi et al. and Choi et al. focus on results obtained using electrical stimulation of the whisker pad, we have focused on the visual stimulation paradigm shown in the supplements accompanying the work of Toi et al. (Fig. S26inToi et al. (2022)). In mice, visual stimulation produced a much larger DIANA signal (0.5%) than the whisker pad stimulation results shown in the main text (0.15%). One of the other key differences between our work and that of Toi et al. and Choi et al. is the use of rats instead of mice. Although both rats and mice rely more on their other senses than on their sense of sight, rats possess higher visual acuity (Prusky et al., 2000). One final difference with the work of Toi et al. was the use of continuous anesthesia infusion. To best approximate the workflow of Toi et al, we gave the animals brief (~16 s) breaks from the stimulus, that is, alternating between measurements with and without stimulus. Alternating between DIANA measurements with and without stimulus also allowed us to simultaneously monitor functional activity based on hemodynamic signal changes.

The observation of a neurovascular signal component in the DIANA data was not a surprise. It is well known that a short ISI drives the hemodynamic response into a steady state (Hodono et al., 2022;Lewis et al., 2016). However, it takes several seconds to reach the hemodynamic steady state. When measurements are collected one scan at a time (as was done byToi et al. (2022)), or alternating measurements with and without stimulus (as was done here), a slow, but large, transient hemodynamic response is ‘titrated’ into each DIANA measurement. If the stimulus paradigm was kept on during consecutive measurements, it might have been possible to think of the steady-state hemodynamic response as a baseline signal shift. Interestingly, supplementary data collected with the stimulus on in 30 consecutive measurements suggest the signal can remain unstable for at least ~20 measurements (Fig. S3).

Given the presence of a hemodynamic background signal variation, one may question if it is better to attempt DIANA at lower field strengths. Neurovascular signal variations are shaped by a combination of BOLD, CBV, and CBF. The short TR in a DIANA measurement saturates the magnetization of the stationary spins considerably. In single-slice measurements with a short echo time, one would expect CBV and CBF to dominate over BOLD signal changes. Both CBV and CBF are field strength independent, explaining why the observed signal changes at 7.0 and 17.2 Tesla were relatively similar (Fig. 5). Therefore, the boost in SNR provided by the stronger magnetic field should outweigh the penalty incurred by a slightly larger hemodynamic background signal. Moreover, these vascularly driven signal changes evolve over much longer timescales than the expected DIANA signal. Simulations showed that these hemodynamic signal variations do not introduce variations in the trial averaged DIANA signal (Fig. 9). When the ISI is short, slow signal variations are effectively removed using the linear drift correction in the post-processing pipeline.

If these ‘slow’ hemodynamic signal variations have a minimal impact on the detectability of the DIANA signal, then why were we unable to detect it? Does the experimental setup allow detecting such small signal variations using this sequence? DIANA experiments use SPGRE sequence with a relatively simple modification (first proposed bySilva & Koretsky (2002)). SPGRE sequences rely on an adequate combination of radiofrequency and gradient spoiling to suppress stimulated echoes and produce a stable signal. To validate this aspect of the setup, we experimentally measured the gradient timing on an oscilloscope, which showed the timing was stable within our measurement accuracy (TR = 5150+/-5 μs). Although the measured TR is slightly longer than the TR specified in the protocol, the fact that it is constant should ensure a stable signal. In addition, we performed phantom experiments to validate that the MRI signal was indeed stable and free of artifacts (Fig. S4). Because the visual stimulus was triggered from the sequence, synchronization was guaranteed even though the effective TR was 150 μs longer than requested.

Yet, we did not detect a DIANA signal. This then raises the question, was our SNR sufficient to detect a DIANA signal? We estimated the tSNR from in-vivo data collected at 17.2 Tesla and used this value to calibrate simulation experiments to investigate the detectability of potential DIANA signals. Interestingly, simulations suggested that at 17.2 Tesla a 0.5% DIANA signal would be easily visible after averaging 16 DIANA measurements (Fig. 6). Arguably, our BOLD derived ROI might include a fair number of voxels biased towards the vascular component some distance from the true area of activation. This bias in our ROI would lead to a reduced effective tSNR. Nevertheless, despite the relatively small number of animals used in this study (four animals per group), our data indicate that with 368 measurements the tSNR was sufficient to detect a ~0.1% signal change. Therefore, even if only one 5^th^of the voxels in our ROI contains neuronally active voxels, we should have been able to detect a DIANA signal.

In general, one could question if swapping the repetition and phase encoding loops is the best strategy to obtain high temporal resolutions in MRI. We observed that signals that are not phase-locked with the paradigm can cause some level of ghosting in image space. Although simulations suggest these ghosts did not impede the detection of a DIANA signal at the site of activation, they might lead to spurious signals in other locations that could prove misleading. Perhaps similar effects also need to be considered in BOLD-fMRI experiments where the repetition loop is placed inside a phase encoding loop (Silva & Koretsky, 2002)? One way to sidestep such challenges is to use Outer Volume Suppression (OVS) or orthogonal refocusing-plane based techniques that forgo phase encoding entirely (Balasubramanian et al., 2021;Morgan et al., 2020;Yu et al., 2014).

It is not clear at what frequency hemodynamic fluctuations become insignificant in the context of a DIANA measurement. In anesthetized animals, these fluctuations depend on the anesthetic and anesthesia level (Cabral et al., 2023). Even in humans, known to have much slower hemodynamic response functions, 0.5 Hz stimuli have been shown to produce signal changes exceeding 0.1% (Hodono et al., 2022;Lewis et al., 2016). Such signals could alias into the DIANA measurement and produce spurious ghost signals in image space. Taking all of this into consideration, we may be hunting for ripples of neuronal activity in a sea of hemodynamic waves.

Unraveling the inner workings of the brain is one of the major objectives in modern science. Advances in MR technology, including extreme magnetic field strengths and cryo-cooled detectors, allowed the development of BOLD fMRI protocols offering unprecedented spatio-temporal resolutions (Abe et al., 2019;Gil et al., 2021;Jung et al., 2021;Yu et al., 2014). Non-BOLD fMRI approaches, such as functional diffusion MRI (Darquié et al., 2001) and functional MR spectroscopy (Prichard et al., 1991), initially proposed more than two decades ago, have also seen significant developments benefiting from the SNR boost offered by ultra-high-field systems (Bihan et al., 2021;Nunes et al., 2021;Stanley & Raz, 2018).

The DIANA technique, with temporal resolutions of only several milliseconds, promised to surpass the capabilities of all the aforementioned methods. A non-invasive measurement technique offering direct observations of neuronal activity with such high temporal resolution would offer huge opportunities for scientific discovery, especially in humans where invasive techniques are generally not possible (Kerkoerle & Cloos, 2022). Unfortunately, translation of DIANA to humans has proven challenging (Hodono et al., 2023), and failure to reproduce the original work in mice has tempered enthusiasm for this technique (Choi et al., 2023). Our efforts to observe DIANA signals in rats echo these observations. Nevertheless, as advances in MR technology continue to unfold, we hope that opportunities to measure neuronal activity more directly will one day emerge.

## Supplementary Material

Supplementary Material

## Data Availability

Data and code are available upon reasonable request.
