# Supplementary material for: No observation of DIANA signals in rats at 7.0 and 17.2 Tesla
Source: Imaging Neurosci (Camb). 2024 Apr 18;2:imag-2-00136. doi: 10.1162/imag_a_00136 (PMC12247614; doi:10.1162/imag_a_00136)
Supplement: Supplementary Material [file imag_a_00136-supp.pdf]

Supplementary material for:

No observation of DIANA signals in rats at 7.0 and 17.2 Tesla

Martijn A. Cloos, Erwan Selingue, Shota Hodono, Romain Gaudin and Luisa Ciobanu

Table S1: Scans per subject

		7.0 T	17.2 T		
		**DIANA**	**DIANA**	**SPGRE***	**EPI***
	**PHANTOM**	6 scans	6 scans		
medetomidine	**Rat 1**	23 scans			
medetomidine	**Rat 2**	23 scans			
medetomidine	**Rat 3**	23 scans			
medetomidine	**Rat 4**	24 scans	25 scans		
medetomidine	**Rat 5**		22 scans		
medetomidine	**Rat 6**		24 scans	23 scans	5 scans
medetomidine	**Rat 7**		21 scans		
ketamine /xylazine	**Rat 8**		22 scans		
ketamine /xylazine	**Rat 9**		22 scans		
ketamine /xylazine	**Rat 10**		22 scans		
ketamine /xylazine	**Rat 11**		22 scans		

*Scans performed to investigate hemodynamic signal changes using a 10 ms stimulus and 190 ms ISI. Not to be confused with the functional localizers used to optimize the slice position and BOLD based ROI.

Table S2: Average tSNR/sqrt(time) across the ROI per measurement

	Stimulus off		Stimulus on	
	**BOLD ROI**	**Control ROI***	**BOLD ROI**	**Control ROI***
**7.0 Tesla**	85 ± 25	52 ± 11	86 ± 23	52 ± 10
**17.2 Tesla**	211 ± 41	139 ± 32	213 ± 41	137 ± 32

* Control ROIs were further from the coil.

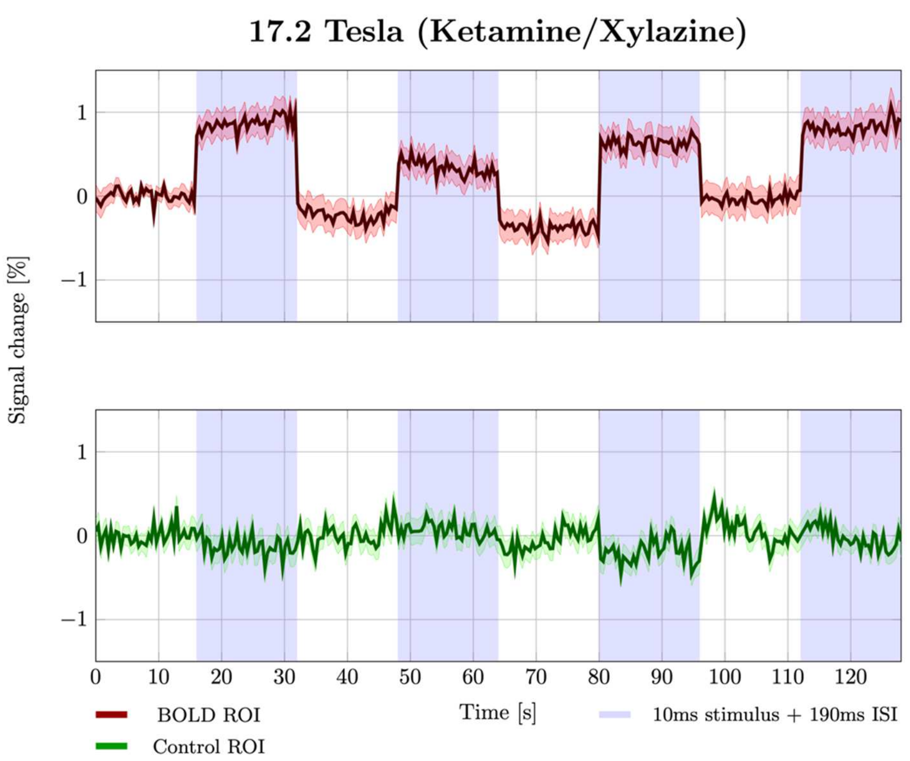

Figure S1: Average signal evolution observed throughout all the scans using Ketamine/Xylazine. Each scan contained 8 subsequent DIANA measurements (128 s of data). The stimulus was on during even numbered measurements only. Solid lines indicate the mean signal across measurements. Shaded areas indicate the 99% confidence interval. Compared to experiments using medetomidine (Figure 5 in the main text) the signal change was slightly smaller.

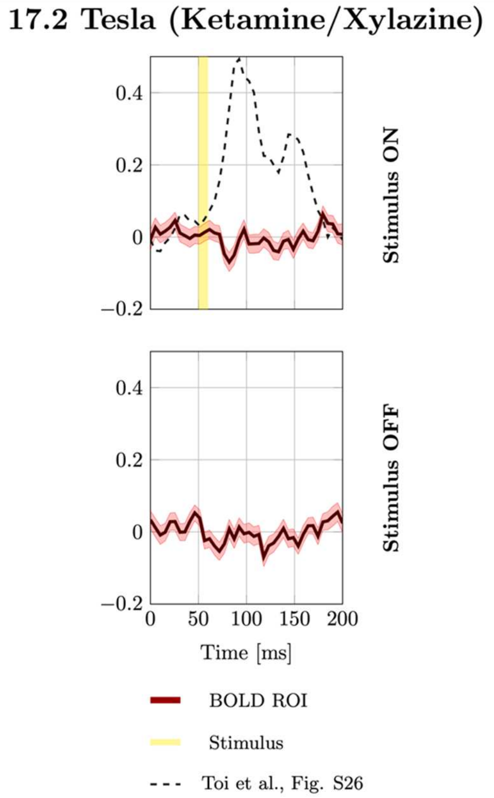

Figure S2: Trial averaged signal evolution observed in the BOLD ROI in experiments using Ketamine/Xylazine. The plots contain data from 4 rats. In total 368 measurements were averaged. Solid lines indicate the mean signal across measurements. Shaded areas indicate the 99% confidence interval. The dashed line shows the expected DIANA signal based on Fig. S26 in Toi et al. The top panel shows the signal measured with the stimulus on. The yellow vertical line represents the stimulus. The bottom panel shows the signal measured without stimulus.

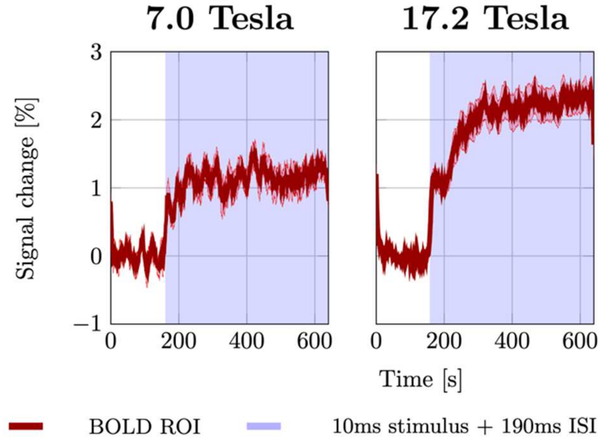

Figure S3: DIANA throughout a long scan starting with 10 measurements without stimulus followed by 30 consecutive measurements with the stimulus on. When the stimulus is turned on the signal jumps up by ~1%, similar to the change seen when alternating measurements (Figure 4). However, the signal did not stabilize, instead the signal increases further over the next ~20 measurements (most noticeable at 17.2 Tesla). Solid lines indicate the mean signal across simulated measurements. Shaded areas indicate the 99% confidence interval.

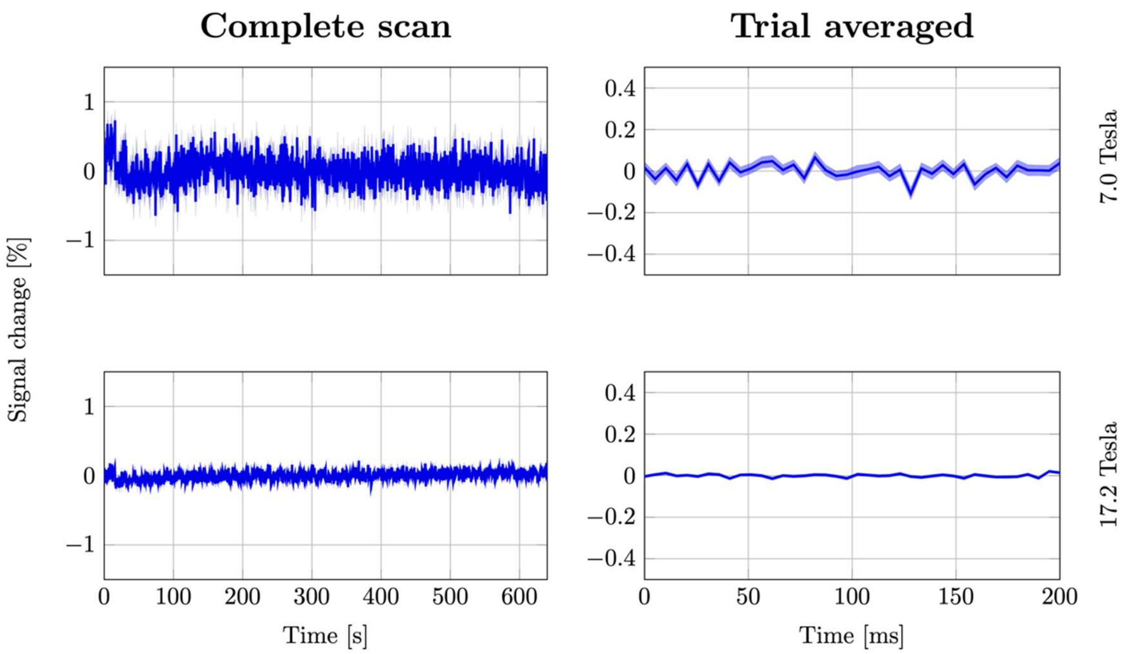

Figure S4: DIANA data collected at 7 and 17.2 Tesla using a phantom. To investigate the stability of the signal, DIANA experiments were performed in a phantom. The left column shows the detrended signal evolution throughout a 640 s DIANA scan containing 40 subsequent DIANA measurements. At both 7 and 17.2 Tesla stable signals were obtained. As expected, the SNR at 7 Tesla was substantially lower than at 17.2 Tesla. The right column shows the trial averaged signals. At 7 Tesla the extra noise produced a wider confidence interval and more noticeable variations in the mean signal. Neither the 7 Tesla nor the 17.2 Tesla data showed signs of artifacts that could lead to the formation of a signal peak that could be mistaken for a DIANA signal. Solid lines indicate the mean signal across measurements. Shaded areas indicate the 99% confidence interval.
